# Spectroscopic Characterisation of the Naphthalene Dioxygenase from *Rhodococcus* sp. Strain NCIMB12038

**DOI:** 10.3390/ijms20143402

**Published:** 2019-07-11

**Authors:** Maria Camilla Baratto, David A. Lipscomb, Michael J. Larkin, Riccardo Basosi, Christopher C. R. Allen, Rebecca Pogni

**Affiliations:** 1 Department of Biotechnology, Chemistry and Pharmacy, Via A. Moro 2, 53100 Siena, Italy; 2 Consorzio per lo Sviluppo dei Sistemi a Grande Interfase (CSGI), via della Lastruccia 3, 50019 Sesto Fiorentino, Italy; 3 School of Biological Sciences, Queen’s University Belfast, 19 Chlorine Gardens, Belfast BT9 5DL, UK

**Keywords:** naphthalene dioxygenase, rieske-type cluster, electron paramagnetic resonance (EPR), spin state, peroxide shunt

## Abstract

Polycyclic aromatic hydrocarbons (PAHs), such as naphthalene, are potential health risks due to their carcinogenic and mutagenic effects. Bacteria from the genus *Rhodococcus* are able to metabolise a wide variety of pollutants such as alkanes, aromatic compounds and halogenated hydrocarbons. A naphthalene dioxygenase from *Rhodococcus* sp. strain NCIMB12038 has been characterised for the first time, using electron paramagnetic resonance (EPR) spectroscopy and UV-Vis spectrophotometry. In the native state, the EPR spectrum of naphthalene 1,2-dioxygenase (NDO) is formed of the mononuclear high spin Fe(III) state contribution and the oxidised Rieske cluster is not visible as EPR-silent. In the presence of the reducing agent dithionite a signal derived from the reduction of the [2Fe-2S] unit is visible. The oxidation of the reduced NDO in the presence of O_2_-saturated naphthalene increased the intensity of the mononuclear contribution. A study of the “peroxide shunt”, an alternative mechanism for the oxidation of substrate in the presence of H_2_O_2_, showed catalysis via the oxidation of mononuclear centre while the Rieske-type cluster is not involved in the process. Therefore, the ability of these enzymes to degrade recalcitrant aromatic compounds makes them suitable for bioremediative applications and synthetic purposes.

## 1. Introduction

Polycyclic aromatic hydrocarbons (PAHs) arise from diverse sources, including petrochemical products and the combustion of fossil fuels [1]. They are a major cause of concern as anthropogenic pollutants in the environment because many are recalcitrant due to of the health hazards associated with them. The PAH naphthalene is released into the environment as coal tar and coal tar products such as creosote [2], and bacteria which degrade naphthalene are widely distributed in nature [3].

It has been found [4,5] that *Rhodococcus* spp. have an important role in the degradation of these substances. Furthermore bacteria from the genus Rhodococcus metabolise a wide variety of pollutants including alkanes, aromatic compounds and halogenated hydrocarbons [6]. The catalytic process is mediated by the Rieske nonhaem iron oxygenases (ROs), such as 1,2-dioxygenase (NDO), in which dioxygen is cleaved and both atoms are inserted across a double bond of an aromatic nucleus to yield a *cis*-dihydrodiol. So the ability of these enzymes to degrade recalcitrant aromatic compounds, makes them suitable for bioremediation applications and for their enantio- and regiospecificity for synthetic purposes [7,8]. Because of their versatility, ROs enzymes have been targeted as platforms for large-scale biosynthesis of aromatic compounds, such as derivatives of indene [9], for the generation of chiral precursors, the synthesis of drugs and biodegradative applications [10].

**Scheme 1:**
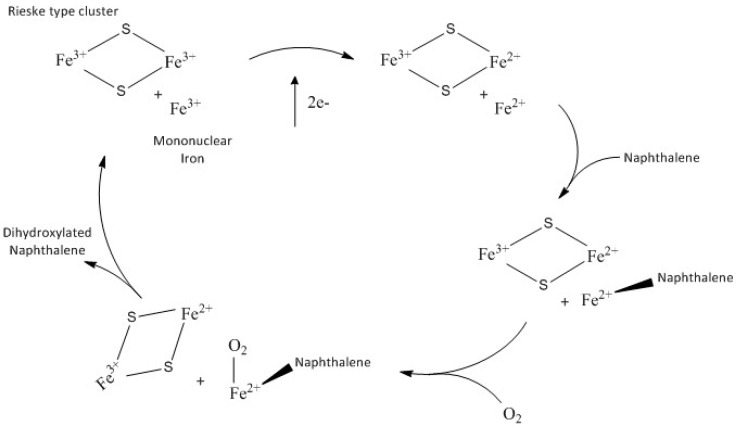
Catalytic cycle of dioxygenases.

ROs have a α_3_β_3_ “mushroom-shaped” quaternary structure with a three-fold structure. The α-subunit can be divided into the Rieske [2Fe-2S] cluster domain and the mononuclear iron-containing domain which has the functional role. The Rieske cluster accepts electrons from the reductase or ferredoxin and transfers them to the mononuclear iron; the catalytic reaction requires electrons that are derived from the NADPH and are transferred by an iron–sulphur flavoprotein to a Rieske-type iron sulphur centre in a ferredoxin protein. The electrons are then passed to the catalytic site of the enzyme (NDO) to be used in the catalysis. The β-subunit does not contain active site residues and its role is believed to be purely structural [7,10]. In all the known structures of ROs the Rieske cluster and the mononuclear iron within a single α-subunit are too far apart for electron transfer (~45Å); however, the quaternary arrangement of the monomers places the Rieske cluster and the mononuclear iron of separate subunits within ~12Å, which is a reasonable distance for electron transfer. This allows room for a “bridge” between the Rieske cluster of one unit and the iron active site. The Rieske cluster is connected to the neighbouring subunit mononuclear iron—believed to be the oxygen site activation [11]—through a conserved aspartate which is shown to be involved in gating electron transport [10]. The crystal structure of free and substrate-bound forms of naphthalene dioxygenase from *Rhodococcus* sp. strain NCIMB12038 was reported previously [12]. A comparison with the crystal structure of naphthalene dioxygenase from *Pseudomonas* sp. [13] showed many possible similarities in substrate specificity between the two enzymes [12]. Furthermore the crystal structure showed that the substrate indole binds near the mononuclear iron suggesting that this is the site for the oxygen bonding [14]. To perform the natural cycle the enzymatic system consists of NADPH reductase that contains FAD and a [2Fe-2S] cluster; an electron transfer protein, NDF, with a Rieske-type cluster; and an oxygenase unit (NDO). The overall reaction stoichiometry requires two electrons and an oxygen molecule to hydroxylate the naphthalene substrate. The general accepted catalytic cycle for dioxygenases [7], is shown in the Scheme 1.

Recently [15] it has been reported that the Fe(II) mononuclear iron and the one-electron reduced Rieske cluster are able to catalyse the dihydroxylation of naphthalene in the absence of NDR and NDF in a single turnover reaction [7]. Naphthalene and molecular oxygen react with NDO to form an amount of product that is stoichiometric with the concentration of the mononuclear Fe(II) sites.

In this paper the naphthalene dioxygenase from *Rhodococcus* sp. strain NCIMB12038 has been studied through electron paramagnetic resonance (EPR) spectroscopy and UV-Vis spectrophotometry. The contemporary use of both techniques allowed characterisation either at room or helium temperatures. Different iron contributions of the enzyme could be elucidated in the native state and under reducing conditions in the presence of sodium dithionite and with O_2_-saturated naphthalene solution. To our knowledge, this is the first report of UV spectra and EPR spectroscopy data for this particular enzyme. The “peroxide shunt”, that is an alternative mechanism for the oxidation of substrate, was also tested. The chromophore of our oxygenase shares common characteristics with other Rieske oxygenases whose catalytic sites have been recently characterised [16,17].

## 2. Results and Discussion

**Figure 1:**
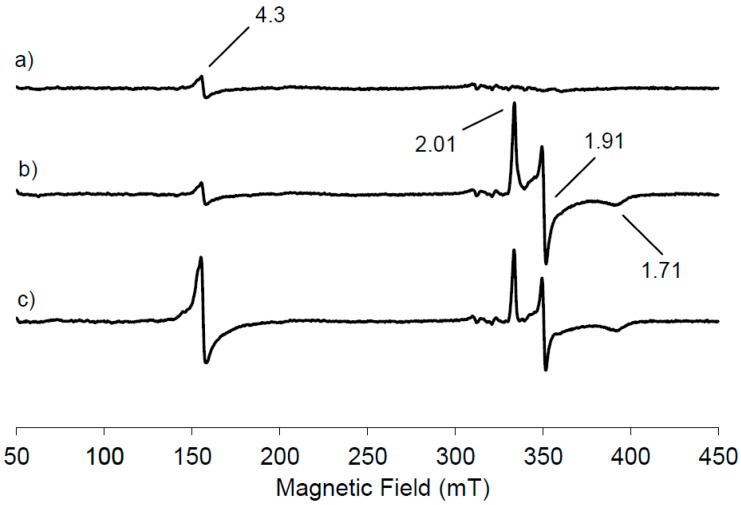
Low temperature (20K) electron paramagnetic resonance (EPR) spectra of naphthalene 1,2-dioxygenase (NDO) samples collected after 10s before freezing in liquid nitrogen: (**a**) in the native state, (**b**) reduced by sodium dithionite and (**c**) reduced NDO after treatment with O_2_-saturated naphthalene. All spectra were recorded at 9.38 GHz, 1mT modulation amplitude, 2 mW microwave power and 100 KHz modulation frequency.

Figure 1a shows the low temperature EPR spectrum of NDO in the native state.

A small resonance is seen at *g* = 4.3 due to the presence of mononuclear high spin Fe(III) contribution, suggesting that most of the mononuclear iron in the resting state of NDO is in the Fe(II) state. This EPR signal has its origin from ferric ion bound in the active site of NDO because the linewidth from peak to peak is 2.5 mT, narrower than that observed from adventitiously bound ferric ion, often present as impurity in protein samples. Furthermore no anisotropic contribution with a *g* < 2 has been observed, indicating that the Rieske cluster is in its diferric state. Therefore from the analysis of the EPR spectrum reported in Figure 1a, the NDO in its resting state contains an oxidised Rieske cluster, which is EPR-silent, due to the antiferromagnetic coupling and a partially reduced mononuclear iron centre. Following the treatment of NDO with the reducing agent dithionite, a new EPR signal appears in the g region around 2 originating from the reduction of the Rieske-type cluster (Figure 1b). It is evident that the signal intensity at *g* = 4.3 due to the mononuclear Fe(III) contribution is unchanged following the appearance of the new EPR signal. One electron reduction of the [2Fe-2S] unit induces the antiferromagnetic exchange interactions of the *S* = 5/2 and *S* = 2 iron sites to yield the *S* = 1/2 system that generates the EPR signals at *g* = 2.01, 1.91, 1.71, centred around *g_av_* = 1.87. Our data are in agreement with those previously reported for NDO from *Pseudomonas* sp. [7,15]. Proteins containing the [2Fe-2S] clusters exhibit this characteristic EPR signature, formed of one *g*-value just above *g* = 2 (range: 2.01–2.05) and two *g*-values below *g* = 2 typically between 1.85 and 1.95. These signals are the results of the exchange interactions *H = J**S*****_1_**⋅***S*_2_** between high spin Fe(III) and high spin Fe(II) site: the *S* = 1/2 ground state of the coupled pair [18]. To get more information on the involvement of the different iron sites in the substrate oxidation, the reduced NDO was reacted with O_2_-saturated naphthalene solution and the EPR spectrum was recorded (Figure 1c). The Rieske-type cluster signal is reduced in intensity of 21% within 10 s with the concomitant increase of 79% in intensity of the mononuclear Fe(III) signal at *g* = 4.3, representing the middle Kramer’s doublet of the high spin Fe(III) in a rhombic environment.

**Figure 2:**
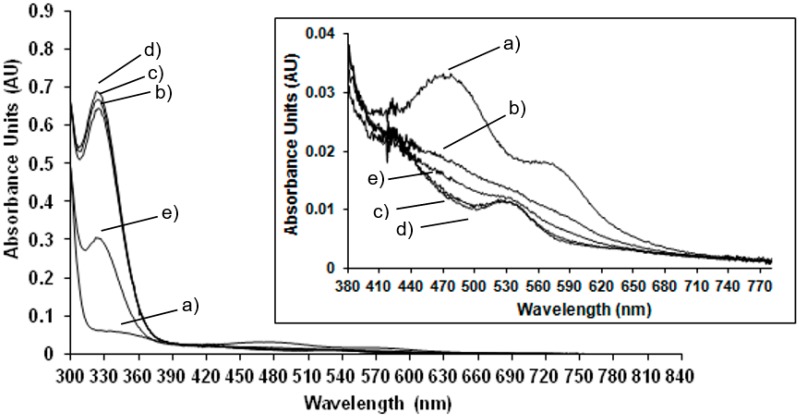
Room temperature UV-Vis spectra of (**a**) native NDO; (**b**) reduced NDO immediately after the addition of sodium dithionite (1.25 mM), t0 min; (**c**) t1 min; (**d**) t5 min; and (**e**) t10 min. In the inset: magnification of the spectra in the range of 380 to 800 nm.

Room temperature spectrophotometric measurements have been performed to compare EPR results carried out at low temperature to those obtained at room temperature, in order to confirm the behaviour of the enzyme at physiological conditions. In Figure 2 the UV-Vis spectrum of the NDO in the native state is compared to the spectra of the system after the addition of the reducing agent, sodium dithionite after different lag times.

In the native state (Figure 2 trace a) the predominant S-Fe(III) charge transfer contribution results in a very broad adsorption band in the visible and near-UV region: maxima at 340 nm and 470 nm and a shoulder at 570 nm are detected (the wavelength region of 380 to 800 nm is magnified in the inset of Figure 2). These absorbance bands are characteristic of the Rieske-type iron in the oxidised state of the protein [19]. After reduction with sodium dithionite the absorbance maxima at 470 nm and 570 nm disappeared and a new maximum appears at 530 nm (see inset of Figure 2). These results are in agreement with previously reported spectrophotometric data for NDO from *Pseudomonas* sp. [19] and observations where spectra maxima were noted, but not previously presented, for NDO from *Rhodococcus* sp. strain NCIMB12038 [20]. As reported in Figure 2 and magnified in the inset, the spectra have been monitored immediately after the addition of sodium dithionite and after 1 min in 2c, 5 min in 2d and 10 min in 2e showing that after 5 min the total reduction of the Rieske-type iron is completed. After 10 min the Rieske-type contribution is no more present, suggesting a new change in the oxidation state of iron.

**Figure 3:**
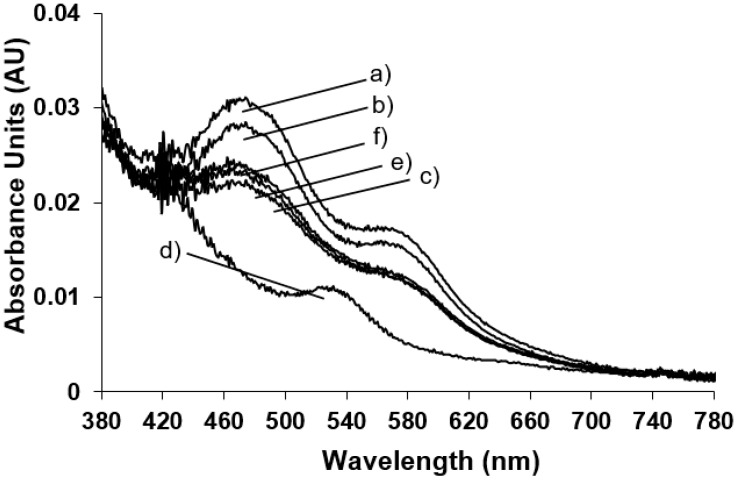
Room temperature UV-Vis spectra of (**a**) native NDO; (**b**) in the presence of O_2_-saturated naphthalene (160 μM); (**c**) the same sample reported in (**b**) reduced by the addition of sodium dithionite (1.25 mM), t0 min; (**d**) t5 min; (**e**) t10 min; and (**f**) t15 min.

The spectrophotometric analysis of the enzyme in its oxidised state have been also performed after bubbling O_2_ into the saturated naphthalene NDO solution (Figure 3 trace b) and the protein in its native state (Figure 3 trace a). The effect of addition of substrate is evident only after the action of the reducing agent that reduces a Fe(III) in the Rieske-type, breaking the antiferromagnetically coupling of the two metal centres and reducing the mononuclear Fe(III) where the substrate is hydroxylated. Therefore the system (NDO plus naphthalene) was reduced with sodium dithionite and monitored after different times from the dithionite addition (Figure 3 traces c–f).

It is interesting to point out that after 5 min (Figure 3, trace d) the Rieske-type iron reduction is completed with the formation of the new absorbance band at 540 nm and with the cycle back of the enzyme at the resting state; after the naphthalene hydroxylation with the restoration of the oxidised Rieske-type iron absorbance bands (Figure 3, traces e and f).

Our data are in agreement with the previously reported analysis showing that NDO with both metal centres in the reduced form is able in a single turnover to yield the *cis*-diol product in the absence of NDR and NDF components [7]. Furthermore the addition of H_2_O_2_ to NDO allows the enzyme to react directly with the naphthalene moiety to rapidly yield the dihydroxylated product in a “peroxide shunt” reaction where the mononuclear Fe(II) is oxidised by hydrogen peroxide and the Rieske-type cluster is ruled out remaining in its oxidised state. The peroxide shunt reaction was completed in less than 10 sec, which is the minimum manual freezing time necessary to perform the EPR measurements in our experiments [8].

**Figure 4:**
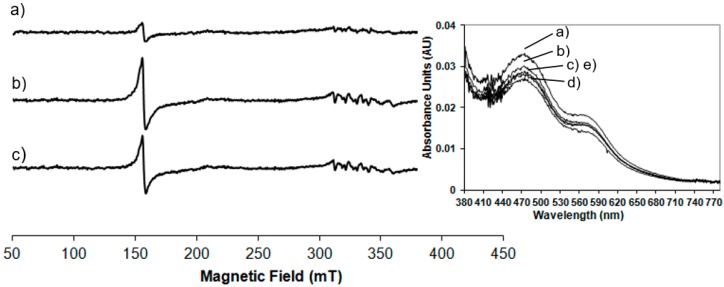
Low temperature (20K) EPR spectra of NDO samples, collected after 10s before freezing in liquid nitrogen: (**a**) mixed with an equal volume of O_2_-saturated naphthalene before and (**b**) after treatment with H_2_O_2_. (**c**) 20K EPR spectrum of NDO, in the absence of naphthalene, treated with H_2_O_2_. All spectra were recorded at 9.38GHz; 1mT modulation amplitude; 2mW microwave power and 100KHz modulation frequency. In the inset: room temperature UV-Vis spectra of (**a**) native NDO; (**b**) NDO after the addition of H_2_O_2_, t0 min; (**c**) t1 min; (**d**) t5 min; and (**e**) t10 min.

In order to test the behaviour of NDO in the presence of hydrogen peroxide, 20K EPR measurements have been performed. In Figure 4a,b the native NDO mixed with an equal volume of O_2_-saturated naphthalene, respectively before and after treatment with hydrogen peroxide are shown. In Figure 4c the EPR spectrum of NDO, in the absence of naphthalene, after the addition of H_2_O_2_ is reported for comparison.

The addition of H_2_O_2_ leads to a rapid increase in the EPR signal at *g* = 4.3. This signal arises from a *S* = 5/2 Fe(III) species with rhombic electronic symmetry (line width = 2.5 mT) at the same time scale of the product formation (<10 s). The oxidation by hydrogen peroxide alone occurs on the mononuclear Fe(III) centre (Figure 4c) as it happens when it is added to the O_2_-saturated naphthalene treated system and the effect is identical to that of mononuclear Fe(III) previously detected and characterised in the NDO turnover in the presence of O_2_-saturated naphthalene and dithionite as reported in Figure 1c where the main effect is on the signal at *g* = 4.3. It is evident that H_2_O_2_ catalyses the oxidation of the mononuclear centre, while the Rieske-type cluster is not involved in the peroxide shunt, as no EPR signal is detected from the reduced Rieske cluster. Figure 4c displays the spectrum of NDO after the reaction with H_2_O_2_ in the absence of naphthalene showing that a smaller percentage of the mononuclear Fe(II) is oxidised. This peroxide shunt pathway represents an alternative mechanism for the oxidation of substrate to the *cis*-diol product. In parallel with the EPR measurements spectrophotometric data are recorded at room temperature during the peroxide shunt and the trend of absorbance maxima monitored at different times is shown in the inset of Figure 4. The spectra show the same absorbance bands at 470 and 570 nm of the Rieske-type in its resting state confirming that this site is not affected by the addition of hydrogen peroxide and ruling out any roles in the oxidation of substrate in the peroxide shunt pathway.

The results indicate that NDO, with reduced Rieske and mononuclear iron centres, is able to react with naphthalene in the absence of the other protein components of the enzyme system. The behaviour found in the NDO from *Rhodococcus* strain NCIMB12038 is similar to that reported for the NDO from *Pseudomonas* previously described [7]. The peroxide shunt followed by EPR measurements is also in agreement to the results published in [16,17] where a Fe(III)-hydroperoxo species or a Fe(III)-superoxo intermediate are formed. Furthermore, the peroxide shunt pathway has shown that only the mononuclear iron site is involved in the oxidation of substrate pointing out a new fast way to get the dihydroxylated compounds avoiding the use of the complex proteins.

## 3. Conclusions

Naphthalene 1,2-dioxygenase (NDO) from *Rhodococcus* sp. has been structurally characterised and studies on the enzyme’s stability performed previously. In the present work, the enzyme was both spectrophotometrically (UV-Vis) and spectroscopically investigated through the application of EPR analysis. Mononuclear high-spin Fe(III) and Rieske-type cluster contributions have been characterised in the presence of the reducing agent, dithionite, and after the O_2_-saturated naphthalene was added. It was concluded that the substrate was dihydroxylated at the mononuclear Fe(III) centre during the peroxide shunt. NDO may be used for bioremediative applications due to its ability to degrade recalcitrant compounds. Despite being distantly related, the enzyme behaves mechanistically in a very similar fashion to its distantly related *Pseudomonas putida* equivalent, where the deduced amino acid sequences have only 33% and 29% identity with the corresponding subunits in *Pseudomonas putida* NCIB 9816-4 [20]. However, the advantage of its increased stability makes it more amenable to further experimentation and mechanistic research.

## 4. Materials and Methods

### 4.1. Chemicals

Sodium dithionite, naphthalene 98%, hydrogen peroxide 30% and absolute ethanol were purchased from Sigma-Aldrich-Fluka and used without further purification.

### 4.2. Enzyme Production and Purification

*Rhodococcus* sp. strain NCIMB12038 cells were grown on naphthalene as the sole carbon and energy source described earlier [21]. The NDO assay used was based on that of Ensley et al. [22], but with 5.5 mM NADH used for assays. The NDO designated as the ISPNAR was purified from naphthalene grown cells as described earlier [20].

### 4.3. Electron Paramagnetic Resonance (EPR) Measurements

EPR solutions were prepared with a final concentration of 39 μM and 26 μM NDO, 2.5 mM and 1.6 mM sodium dithionite, respectively, in 50 mM TEG buffer (50 mM Tris-HCl (pH 7.8) containing 10% ethanol, 10% glycerol and 0.5 mM dithiothreitol). NDO was in the oxidised state and the reduction of the enzyme was obtained by addition of oxygen-free buffered sodium dithionite to NDO. O_2_-saturated naphthalene was first dissolved in ethanol and the final concentration of 250 μM and 166 μM was reached using TEG buffer. Reactions with peroxide were carried out using 39 μM, 26 μM and 19.5 μM final concentrations of enzyme, naphthalene 250 μM, 166 μM and 125 μM, respectively, and H_2_O_2_ added at the end with final concentrations of 250 mM, 166 mM and 125 mM. The reactions were stopped by rapid immersion of the EPR tube in liquid nitrogen within 10 s from the addition of the last reactant.

CW-X-band (9GHz) EPR measurements were performed with a Bruker E500 Elexsys Series using the Bruker ER 4122SHQE cavity and the Oxford helium continuous flow cryostat (ESR900).

Systems containing [2Fe-2S] in the resting state are diamagnetic, indicative of antiferromagnetic coupling between two Fe(III) ions to give *S* = 0 ground state. Reduced forms are paramagnetic with a spin = 1/2 ground state, which gives rise to slightly anisotropic EPR spectra and *g*_av_ = 1.87. In this case the antiferromagnetic exchange interactions, between high-spin Fe(III) (*S* = 5/2) and high-spin Fe(II) (*S* = 2), gives rise to an *S* = 1/2 ground state. The total spin Hamiltonian equation for the system is reported below.

$$H=-JS_{1}\cdot S_{2}+\beta S_{1}\cdot g_{1}\cdot B+\beta S_{2}\cdot g_{2}\cdot B+D_{1}S_{1z}^{2}+D_{2}S_{2z}^{2}$$

In this particular context *S*_1_ = 5/2 and *S*_2_ = 2 [18,23].

### 4.4. Spectrophotometric (UV-Vis) Measurements

Spectrophotometric spectra have been recorded at room temperature using a UV-Vis Perkin Elmer Lambda 900 spectrophotometer.

Solutions for spectrophotometric experiments were prepared with a final concentration of 19 μM NDO, 1.25 mM sodium dithionite in 50mM TEG buffer (50mM Tris-HCl (pH 7.8) containing 10% ethanol, 10% glycerol and 0.5 mM dithiothreitol) recorded at different times: 0, 1, 5 and 10 min from the addition of dithionite.

The reactions in the presence of O_2_-saturated naphthalene and then reduced by the addition of sodium dithionite (1.25 mM) were carried out with a final concentration of enzyme 19 uM, 160 uM of naphthalene and 1.25 mM of sodium dithionite. The reactions were monitored at different times: 0, 1, 5 and 10 min from the addition of dithionite.

The peroxide shunt was followed spectrophotometrically at a final concentration of NDO 19 uM and 122 mM hydrogen peroxide recorded at 0, 1, 5 and 10 min from the addition of H_2_O_2_.
